# Cross-Sectional and Longitudinal Associations between Egg Consumption and Metabolic Syndrome in Adults ≥ 40 Years Old: The Yangpyeong Cohort of the Korean Genome and Epidemiology Study (KoGES_Yangpyeong)

**DOI:** 10.1371/journal.pone.0147729

**Published:** 2016-01-25

**Authors:** Hye Won Woo, Bo Youl Choi, Mi Kyung Kim

**Affiliations:** 1 Department of Preventive Medicine, College of Medicine, Hanyang University, Seoul, Korea; 2 Institute for Health and Society, Hanyang University, Seoul, Korea; College of Medicine, National Taiwan University, TAIWAN

## Abstract

Since the 1970s, the public has been advised to limit egg consumption even though there is little evidence of any harmful effect of eggs on blood cholesterol. The purpose of this cross-sectional and prospective study was to evaluate the potential association between egg consumption and metabolic syndrome (MetS) and MetS components in adults ≥ 40 years in KoGES_Yangpyeong. Yangpyeong is a rural area in South Korea. A total of 2,887 subjects (men 1,115, women 1,772) were recruited from 2005 to 2009, based on a physical examination and questionnaires administered using standardized protocol. After excluding subjects who had MetS at baseline, 1,663 subjects (675 men, 958 women) were followed for 3.20 years (range: 0.34–8.70). During the follow-up period, MetS occurred in 289 subjects. More than 3 eggs per week was significantly associated with decreased risk of MetS in both men (RR = 0.46, 95% CI, 0.26–0.82, *P* for trend = 0.1093) and women (RR = 0.54, 95% CI, 0.31–0.93, *P* for trend 0.0325) compared to non-users. There was a cross-sectional inverse relationship between egg consumption and abdominal obesity in men and women. Also, prospectively, higher egg consumption in men was associated with a decreased risk of high fasting blood glucose (RR = 0.39, 95% CI, 0.22–0.67, *P* for trend = 0.0042) and high triglycerides (RR = 0.42, 95% CI, 0.22–0.80, *P* for trend = 0.1080). In conclusion, our findings suggest that higher egg consumption may reduce the risk of MetS both in men and women, and the risk of high fasting blood glucose and high triglycerides in men. Current guidelines regarding egg consumption may need to be re-visited for healthy middle-aged and elderly people.

## Introduction

Data from epidemiologic studies on coronary heart disease (CHD) have consistently reported a positive association with increased LDL cholesterol [1]. Because egg, which contain about 71% of the recommended daily intake of cholesterol, is a major source of dietary cholesterol, the relationship between CHD and dietary intake of eggs has received much attention [1] and the public has been advised to limit consumption of eggs [2]. However, three US and one Japanese prospective cohort studies did not show any association between egg consumption and CHD, cardiovascular disease (CVD), or mortality in free living adults [3–6]. In a general population-based study, egg consumption was not cross-sectionally correlated with high serum cholesterol concentration [7].

Nevertheless, there has been concern that dietary cholesterol, which may contribute to abnormal lipid profiles by changing apolipoprotein profiles [8], might increase the risk of CVD among diabetic patients. In some previous epidemiologic studies among diabetic patients, harmful effects of egg consumption on CVD incidence, which have not been observed in healthy populations [3–6], were observed. However, a clinical trial feeding a diet high in cholesterol due to egg to diabetic patients did not have any adverse effects on cardiovascular risk factors such as blood lipid profiles or blood glucose profiles [9]. Another clinical trial comparing whole eggs with a yolk-free egg substitute in subjects with metabolic syndrome (MetS) reported superior atherogenic lipoprotein profiles and insulin sensitivity in the group consuming the whole eggs [10]. It was suggested that egg yolk rich in phosphatidyl choline and other phospholipids has a beneficial effect [10].

Until now there has been little prospective evidence on the relationship between egg consumption and cardiometabolic risk factors, particularly in free living population. We, therefore, examined the prospective association between the consumption of eggs as a whole food (as opposed to individual components of eggs such as cholesterol) and the risk of MetS among men and women 40 years or older in the community-based Yangpyeong cohort in Korea.

## Materials and Methods

### Study design and population

Study subjects were members of the Yangpyeong cohort, a **M**ulti-**R**ural community **Cohort** (MRCohort) in the Korean Genome and Epidemiology Study (KoGES), which was established for cardiovascular disease prevention. Yangpyeong County is located 45 km east of Seoul. Subjects were recruited by selecting villages using multistage cluster sampling. District leaders and representatives of women and young adults encouraged residents to participate in the study.

At baseline, 3,183 men and women ≥ 40 years old were enrolled from 2005 to 2009. A total of 296 were excluded for the following reasons: physician-diagnosed coronary artery disease (n = 133), stroke (n = 74), or cancer (n = 61); information about MetS components missing in the baseline survey (n *=* 17); implausible dietary intake (< 500 or > 4000 kcal/d, or > 10 missing food items) (n = 21). Ultimately, 2,887 subjects remained in the cross-sectional analysis. An additional 1,224 subjects with MetS at baseline were excluded, leaving 1,663 subjects in the prospective analysis.

A comprehensive health examination was conducted using standard protocols. We collected information on demographics such as age, marital status, education level, smoking habits, exercise frequency, and alcohol consumption. Height was measured with a stadiometer to the nearest 0.1 cm, and weight was measured with a metric scale to the nearest 0.01 kg while the subjects were wearing light clothing with no shoes. Body Mass Index (BMI) was calculated as weight (kg) /height (m^2^). Waist circumference (WC) was measured halfway between the lowest rib margin and the iliac crest. Blood pressure (BP) was measured after each subject had been sitting for ≥ 5 min. Systolic BP and diastolic BP measurements were recorded at least twice at 5-min intervals, and an average value was used. Blood samples were collected after ≥8 h of fasting, and serum concentrations of total cholesterol, HDL cholesterol (HLD-C), triacylglycerol (TG), and glucose were quantified with an ADVIA1650 Automatic Analyzer (Siemens, New York, USA).

Egg and nutrient intake were estimated from a semi-quantitative food frequency questionnaire (FFQ) consisting of 106 food items (including eggs) with nine frequency categories ranging from ‘never or rarely’ to ‘three times/day’, and three portion sizes specified for each food item. Trained interviewers asked subjects how often, on average, during the previous year they had consumed eggs and how many eggs they had eaten on each occasion (units of consumption were 0.5, 1, and 1.5 eggs). We divided the subjects into 4 categories based on egg consumption (non-users, 0 > to ≤ 1 egg/wk, 1 > to ≤ 3 eggs/wk, > 3 eggs/wk). The validity and reproducibility of the food frequency questionnaire have been examined in detail elsewhere [11]. Total energy intake was calculated using the 2011 nutrient database of the Korean Nutrition Society [12].

### Metabolic Syndrome follow-up

All participants were asked to return to the research center every 2–4 years (average, 2.8 years). Of the 3,183 subjects that participated at baseline, 2,576 and 1,427 subjects returned for a 2^nd^ visit (2007–2013) and 3^rd^ visit (2010–2013), respectively. In the follow-up visits we identified incident cases of MetS using data for five components of MetS, which were obtained according to the same protocol as at baseline,

MetS was defined according to the updated National Cholesterol Education Program Adult Treatment Panel III (NCEP ATP III) criteria, modified according to the International Diabetes Federation and Korean Diabetes Association criteria for fasting plasma glucose [13] and WC [14]. Subjects were diagnosed with MetS if they had three or more of the following five components: 1) abdominal obesity, WC ≥ 90 cm in men and ≥ 85 cm in women; 2) high BP, ≥ 130/85 mm Hg or taking antihypertensive medication; 3) high fasting blood glucose, ≥ 100 mg/dL or taking medication to treat diabetes mellitus; 4) high TG, ≥ 150 mg/L, and 5) low HDL-C, < 40 mg/dL in men and < 50mg/dL in women. MetS is an event with multiple relapses, but the first diagnosis after enrollment was treated as the new incident.

The person-years of follow-up for each participant was calculated from the day of enrollment to the day of diagnosis of MetS, or to the day of death from any cause, or to the day of diagnosis of cardiovascular disease or cancer. The end point of this study was diagnosis of MetS on follow-up. Participants who died from any cause were treated as censored subjects, and those who reported a diagnosis of CVD or cancer between visits were also censored to minimize the potential effect of their treatment on the MetS components. If subjects were lost to follow-up, half the median follow-up time of the subjects who were successfully followed-up was assigned as their follow-up time [15]. If subjects were diagnosed with cardiovascular disease or cancer but did not report the date of diagnosis, we assigned a diagnosis time half-way between the last visit and the most recent visit [15].

The study was conducted in accordance with the Declaration of Helsinki, and the protocol was approved by the Institutional Review Board (IRB) of Hanyang University. All participants provided written informed consent.

### Statistical analysis

Means and standard deviations for continuous variables and percentage for categorical variables were used to describe subject characteristics. Age-adjusted averages or percentages according to egg consumption (non-users, 0 > to ≤ 1 egg/wk, 1 > to ≤ 3 eggs/wk, > 3 eggs/wk) were obtained using a general linear model (GLM) to assess potential confounders. *P-*values for linear trend were obtained by treating the median value of egg consumption in each category as a continuous value. Cross-sectional associations between egg consumption and prevalent MetS at baseline were assessed by logistic regression analysis. We evaluated the prospective association among subjects who did not have MetS at baseline by a modified Poisson regression model using a robust error estimator [16, 17].

When evaluating dietary exposure as a determinant of disease occurrence we used the averages of egg consumption to reduce within-subject variation and represent the long-term diet accurately [18]. Therefore, average egg consumption was calculated as mean egg intake from baseline until occurrence of MetS, and we stopped updating diets in the intervals during which individuals reported a diagnosis of CVD or cancer due to change in diet after development of diagnosis [18]. In cross-sectional and prospective analyses, we presented OR or RR according to egg consumption group (non-users, 0 > to ≤ 1 egg/wk, 1 > to ≤ 3 eggs/wk, > 3 eggs/wk) compared with non-users of eggs. Additionally, the daily 10g increments of egg consumption were introduced as a continuous variable. Variables showing significant linear trends across the egg consumption groups were considered as potential confounders, including age (years), marital status (married or single), educational level ≥ 12 years (yes or no), regular exercise for at least 30 min on three or more days per week (yes or no), smoking status (current or non-smoker), and drinking status (current or non-drinker). We analyzed the interaction effect of average egg consumption with BMI (normal weight; < 23 kg/m^2^ or overweight; ≥ 23 kg/m^2^) on MetS and its components in the multivariable model. All analyses were completed with the use of SAS software (version 9.3; SAS Institute, Cary, NC).

## Results

Table 1 presents the general characteristics of the participants. The average age of the men was 60.9 years, and that of the women was 59.3 years. Men were more likely to be married, well-educated, current smokers and current drinkers and to consume more energy and eggs than women.

**Table 1:** Baseline characteristics of the study population.

Characteristic	Men	Women
*n*	1115	1772
Age (years)	60.9±10.2	59.3±10.5
Married (%)	95.7	78.6
Educational level (≥ High-school, %)^1^	38.8	23.1
Regular exercise (%)^2^	22.2	24.4
Current smoker (%)	32.9	2.9
Current drinker (%)	69.6	32.1
BMI (kg/m^2^)	24.2±3.0	24.8±3.2
Total energy intake (kcal/d)	1766.4±523.4	1472.1±446.4
Egg consumption (No./wk)	2.0±2.8	1.2±1.9

All values are expressed as Mean±SD or percentage.

^1^ ≥ High school graduates (12 years of education).

^2^ ≥ 3 times/week and 30 min/session.

Table 2 shows potential confounding factors according to egg consumption group. Among all subjects in the cross-sectional analysis, mean age decreased and education level increased with egg consumption. In women, BMI decreased slightly (*P* for trend = 0.0215), and intake of total energy, retinol, and cholesterol increased with egg consumption. However, among the variables showing significant linear trends across egg consumption groups, dietary retinol (r = 0.44) and dietary cholesterol (r = 0.65) were highly correlated with egg consumption and thus were not considered as potential confounders. Similar trends were observed in subjects without MetS, except that there was a stronger linear trend for regular exercise in men (*P* for trend = 0.0409) and a weaker trend for BMI in women (*P* for trend = 0.3224). We describe the details of the potential confounding factors in the prospective analysis in Supporting Information (S1 Table).

**Table 2:** Age-adjusted characteristics of the study population, according to egg consumption.

	Weekly egg consumption (No/week)				
	Total subjects *(n = 2887)*				
Variable	0	0–1	1–3	>3	*P* trend^3^
***Men (n)***	*229*	*296*	*320*	*270*	
Age (years)	63.5±0.67	61.3±0.59	59.5±0.56	59.9±0.61	0.0022
Married (%)	95.5	97.0	96.4	93.6	0.0669
Education (≥ High-school, %)^1^	29.1	33.8	45.1	45.2	0.0002
Regular exercise (%)^2^	22.8	18.0	23.6	24.6	0.2162
Current smoker (%)	34.6	31.3	31.4	35.0	0.5298
Current drinker (%)	64.7	71.5	70.5	70.6	0.4924
BMI (kg/m^2^)	24.4±0.19	24.2±0.17	24.1±0.16	23.9±0.18	0.0707
***Daily dietary intake***					
Total energy (kcal/d)	1628.3±31.94	1657.8±27.91	1802.2±26.91	1960.2±29.25	< .0001
Fiber (g/d)	17.4±0.35	16.9±0.30	16.8±0.29	17.1±0.32	0.8790
Retinol (μg/d)	33.4±3.28	41.4±2.87	60.7±2.76	99.6±3.00	< .0001
β-Carotene (μg/d)	2542.6±100.86	2564.9±88.12	2512.7±84.95	2636.8±92.36	0.4186
Vitamin C (mg/d)	79.6±2.61	79.4±2.28	83.5±2.20	82.0±2.39	0.4855
Cholesterol (mg/d)	72.5±6.26	96.6±5.47	145.6±5.27	263.5±5.73	< .0001
***Metabolic syndrome components***					
Waist circumference (cm)	87.9±0.55	87.0±0.48	85.9±0.46	86.4±0.50	0.1288
Fasting glucose (mg/dL)	107.5±1.65	104.9±1.44	104.8±1.39	105.0±1.51	0.5518
Triglycerides (mg/dL)	173.6±7.40	162.9±6.46	163.0±6.23	165.6±6.77	0.7881
HDL cholesterol (mg/dL)	43.0±0.75	43.2±0.66	43.4±0.63	44.1±0.69	0.2534
Systolic blood pressure (mm Hg)	125.2±1.05	124.6±0.92	124.5±0.88	123.9±0.96	0.4101
Diastolic blood pressure (mm Hg)	80.7±0.68	80.5±0.59	80.6±0.57	80.2±0.62	0.5700
***Women (n)***	*595*	*495*	*418*	*264*	
Age (years)	63.0±0.41	59.4±0.45	56.4±0.49	55.4±0.62	< .0001
Married (%)	78.1	78.7	80.2	76.2	0.5728
Education (≥ High-school, %)^1^	16.3	19.0	30.3	34.9	< .0001
Regular exercise (%)^2^	22.9	23.1	26.6	26.4	0.1848
Current smoker (%)	4.3	2.0	2.2	2.4	0.2073
Current drinker (%)	32.8	30.0	33.3	32.7	0.7766
BMI (kg/m^2^)	24.9±0.13	25.1±0.14	24.3±0.16	24.6±0.20	0.0215
***Daily dietary intake***					
Total energy (kcal/d)	1379.7±17.6	1431.5±18.88	1536.3±20.75	1654.6±26.14	< .0001
Fiber (g/d)	14.1±0.28	15.0±0.30	17.3±0.33	19.2±0.41	0.6584
Retinol (μg/d)	29.9±2.03	37.8±2.18	55.5±2.39	85.1±3.02	< .0001
β-Carotene (μg/d)	2303.9±57.87	2317.5±62.08	2302.6±68.22	2363±85.94	0.6126
Vitamin C (mg/d)	77.7±1.70	80.1±1.83	81.6±2.01	82.2±2.53	0.1422
Cholesterol (mg/d)	52.9±3.39	78.6±3.63	124.5±3.99	218.3±5.03	< .0001
***Metabolic syndrome component***					
Waist circumference (cm)	84.7±0.37	85.4±0.39	83.2±0.43	83.4±0.54	0.0023
Fasting glucose (mg/dL)	101.6±0.89	101.4±0.95	99.9±1.04	97.6±1.31	0.0069
Triglycerides (mg/dL)	144.8±3.53	145.7±3.78	141±4.16	140.6±5.23	0.3939
HDL cholesterol (mg/dL)	45.2±0.44	45.0±0.47	45.4±0.52	45.9±0.65	0.3331
Systolic blood pressure (mm Hg)	120.5±0.67	122.7±0.72	122.1±0.79	120.5±0.99	0.7793
Diastolic blood pressure (mm Hg)	78.0±0.42	77.9±0.45	77.3±0.50	77.0±0.62	0.1489

All values were adjusted for age and expressed as Mean±SE or percent.

All nutrients were energy-adjusted except for total energy intake.

^1^ ≥ High school graduates (12 years of education).

^2^ ≥ 3 times/week and 30 min/session.

^3^*P*-values for linear trends were obtained using the general linear model (GLM).

### Association of egg consumption with metabolic syndrome

The prevalence of MetS at baseline was 39.5% in men and 46.0% in women. During an average follow-up of 3.2 years, a total of 289 MetS events occurred and the incidence rate of MetS was 56/1,000 person-years. Multivariable-adjusted models did not greatly change the associations in the age-adjusted models. All three approaches, one cross-sectional analysis at baseline, and two prospective analyses using egg consumption at baseline and average egg consumption, showed similar inverse associations between egg consumption and MetS. However, a significant inverse relationship between egg consumption and MetS prevalence was only observed in the multivariable-adjusted model for men (OR = 0.66, 95% CI, 0.45–0.97 for the highest egg consumption vs. non-users; *P* for trend = 0.0863) and not for women. There was no significant association between MetS and baseline egg consumption in the prospective analysis. Compared with non-use, more than 3 eggs per week was significantly associated with decreased risk of MetS among men (RR = 0.46, 95% CI, 0.26–0.82 for the highest consumption group, *P* for trend = 0.1093) and women (RR = 0.54, 95% CI, 0.31–0.93, *P* for trend = 0.0325) in a multivariable model for the average egg consumption (Table 3). In the analyses of daily 10g increments of egg consumption, there was no linear dose-response relationship with MetS risk among men and it was marginal in significance level among women (p = 0.0504).

**Table 3:** Cross-sectional and prospective associations between metabolic syndrome and egg consumption.*

	Weekly egg consumption (No/week)					Continuous variables
	0	0–1	1–3	> 3	*P* trend^3^	per daily 10 g increment
***Men***						
***Cross-sectional analysis (n = 1115)***						
Prevalent cases at baseline / No. of subjects	97 / 229	116 / 296	124 / 320	103 / 270		
Median intake (min, max)	0	0.58 (0.16, 0.86)	1.50 (1.50, 2.25)	5.25 (3.50, 31.5)		
Age-adjusted OR	1.00	0.83 (0.96–0.99)	0.78 (0.55–1.11)	0.77 (0.53–1.10)	0.2971	0.98 (0.92–1.04)
Multivariable-adjusted OR^1^	1.00	0.80 (0.56–1.15)	0.70 (0.49–1.00)	0.66 (0.45–0.97)	0.0863	0.96 (0.90–1.03)
***Prospective analysis (n = 675)***						
**Egg consumption at baseline**						
No. of cases / person years	28 / 380	28 / 566	30 / 662	24 / 525		
Median intake (min-max)	0	0.58 (0.12, 0.86)	1.50 (1.50, 2.25)	5.25 (3.50, 31.5)		
Age-adjusted RR	1.00	0.66 (0.40–1.08)	0.59 (0.36–0.97)	0.59 (0.35–1.00)	0.2226	0.97 (0.87–1.09)
Multivariable-adjusted RR^2^	1.00	0.68 (0.40–1.13)	0.63 (0.38–1.06)	0.66 (0.38–1.15)	0.4113	0.99 (0.88–1.10)
**Average egg consumption**						
No. of cases / person years	25 / 270	28 / 583	36 / 782	21 / 498		
Median intake (min-max)	0	0.58 (0.06, 0.92)	1.50 (1.04, 2.91)	4.25 (3.04, 31.5)		
Age-adjusted RR	1.00	0.50 (0.30–0.83)	0.46 (0.28–0.76)	0.43 (0.25–0.75)	0.0758	0.94 (0.82–1.08)
Multivariable-adjusted RR^2^	1.00	0.52 (0.31–0.88)	0.49 (0.29–0.81)	0.46 (0.26–0.82)	0.1093	0.95 (0.83–1.10)
***Women***						
***Cross-sectional analysis (n = 1772)***						
Prevalent cases at baseline / No. of subjects	313 / 595	251 / 495	155 / 418	95 / 264		
Median intake (min-max)	0	0.58 (0.12, 0.86)	1.50 (1.50, 2.25)	3.50 (3.50, 31.5)		
Age-adjusted OR	1.00	1.24 (0.88–1.44)	0.73 (0.56–0.96)	0.74 (0.54–1.01)	0.0078	1.01 (0.89–1.03)
Multivariable-adjusted OR^1^	1.00	1.11 (0.84–1.45)	0.85 (0.63–1.15)	0.77 (0.54–1.10)	0.0683	0.90 (0.82–0.99)
***Prospective analysis (n = 958)***						
**Egg consumption at baseline**						
No. of cases / person years	55 / 851	53 / 781	48 / 819	23 / 540		
Median intake (min-max)	0	0.58 (0.12, 0.86)	1.50 (1.50, 2.25)	3.50 (3.50, 31.5)		
Age-adjusted RR	1.00	1.19 (0.83–1.72)	1.13 (0.77–1.66)	0.84 (0.52–1.37)	0.3973	0.95 (0.85–1.05)
Multivariable-adjusted RR^2^	1.00	1.19 (0.83–1.72)	1.10 (0.74–1.62)	0.81 (0.49–1.33)	0.2971	0.94 (0.84–1.04)
**Average egg consumption**						
No. of cases / person years	52 / 617	55 / 876	53 / 986	19 / 512		
Median intake (min-max)	0	0.40 (0.06, 1.00)	1.50 (1.04, 2.88)	3.68 (3.04, 31.5)		
Age-adjusted RR	1.00	0.82 (0.57–1.19)	0.77 (0.53–1.12)	0.56 (0.33–0.96)	0.0453	0.87 (0.76–1.01)
Multivariable-adjusted RR^2^	1.00	0.82 (0.57–1.19)	0.75 (0.52–1.10)	0.54 (0.31–0.93)	0.0325	0.86 (0.74–1.00)

*Values are expressed as odds ratios (OR) or relative risk (RR) and 95% confidence intervals.

^1^Multivariable-adjusted OR: Multivariate logistic regression analysis adjusted for age (years), educational level (≥ 12 years, yes or no) and total energy intake (kcal/d) for men and age (year), educational level (≥ 12 years, yes or no), BMI (kg/m2) and total intake energy (kcal/d) for women.

^2^ Multivariable-adjusted RR: Multivariate Poisson regression analysis adjusted for age (years), educational level (≥ 12 years, yes or no), regular exercise (regular exercise at least 30 min on three or more days per week, yes or no) and total energy intake (kcal/d) for men and age (year), educational level (≥ 12 years, yes or no), and total intake energy (kcal/d) for women.

^3^*P* values for linear trends were obtained by treating the median value of egg consumption in each category as a continuous value.

### Association of egg consumption with metabolic syndrome components

Table 4 shows the association between egg consumption and individual MetS components. Among the MetS components, as compared with the non-users, the highest egg consumption had a cross-sectionally inverse association with abdominal obesity in men (OR = 0.49, 95% CI, 0.33–0.72, *P* for trend = 0.0018) and women (OR = 0.68, 95% CI, 0.44–1.05, *P* for trend = 0.0458). However, these associations appeared in the opposite direction (*P* for trend = 0.0148 using baseline egg consumption) or disappeared (*P* for trend = 0.1385 using average consumption) in the prospective analyses. There was a U-shaped association between average egg consumption and the risk of high blood pressure for both baseline egg consumption and average egg consumption. Baseline egg consumption appeared to reduce the risk of high fasting blood glucose, high TG, and low HDL-C, but these effects were not statistically significant. Average egg consumption also reduced the risk of high fasting blood glucose (RR = 0.39, 95% CI, 0.22–0.67 for more than 3 eggs per week for men, *P* for trend = 0.0042) and of high TG (RR = 0.42, 95% CI, 0.22–0.80, *P* for trend = 0.1080), compared to the non-users. Although we did not observe significant linear trends between egg consumption and low HDL-C, the risk of low HDL-C was lower in the middle group (1 > to ≤ 3 eggs/wk) compared to non-users of eggs. In women, there were no significant linear trends according to egg consumption, although a significant negative association with high TG and low HDL-C was observed in women who consumed 0–1 or 1–3 eggs/wk.

**Table 4:** Multivariable-adjusted ORs, RRs and 95% CIs of individual metabolic syndrome components according to egg consumption group.*

	Weekly egg consumption (No/week)					Continuous variables	
	0	0–1	1–3	> 3	*P* trend^3^	per daily 10 g increment	
***Men***							
**Abdominal obesity** (≥ 90cm)							
Multivariable-adjusted OR^1^ *(n = 1115)*	1.00	0.73 (0.51–1.04)	0.59 (0.41–0.84)	0.49 (0.33–0.72)	0.0018	0.91 (0.85–0.98)	
Egg consumption at baseline RR^2^ *(n = 564)*	1.00	0.60 (0.24–1.53)	0.98 (0.44–2.21)	1.79 (0.80–3.97)	0.0148	1.07 (0.98–1.16)	
Average egg consumption RR^2^ *(n = 564)*	1.00	0.49 0.19–1.32)	0.79 (0.34–1.86)	1.12 (0.46–2.74)	0.1385	1.02 (0.92–1.14)	
**High blood pressure** (≥ 130/85 mmHg)							
Multivariable-adjusted OR^1^ *(n = 1115)*	1.00	0.91 (0.64–1.28)	1.02 (0.72–1.44)	0.86 (0.60–1.24)	0.4462	0.99 (0.93–1.05)	
Egg consumption at baseline RR^2^ *(n = 428)*	1.00	0.49 (0.27–0.88)	0.58 (0.34–0.99)	0.69 (0.40–1.19)	0.9425	0.96 (0.86–1.08)	
Average egg consumption RR^2^ *(n = 428)*	1.00	0.44 (0.25–0.77)	0.44 (0.25–0.76)	0.62 (0.36–1.07)	0.8794	0.98 (0.86–1.11)	
**High fasting blood glucose** (≥ 100 mg/dL)							
Multivariable-adjusted OR^1^ *(n = 1115)*	1.00	1.02 (0.72–1.45)	0.82 (0.58–1.17)	0.88 (0.61–1.28)	0.5052	0.99 (0.93–1.05)	
Egg consumption at baseline RR^2^ *(n = 465)*	1.00	0.93 (0.58–1.50)	1.07 (0.69–1.67)	0.64 (0.38–1.09)	0.0769	0.90 (0.81–1.01)	
Average egg consumption RR^2^ *(n = 465)*	1.00	0.60 (0.38–0.96)	0.63 (0.41–0.97)	0.39 (0.22–0.67)	0.0042	0.85 (0.73–0.98)	
**High triglyceride** (≥ 150 mg/dl)							
Multivariable-adjusted OR^1^ *(n = 1115)*	1.00	0.96 (0.68–1.37)	0.83 (0.58–1.19)	0.87 (0.60–1.26)	0.5220	1.01 (0.95–1.08)	
Egg consumption at baseline RR^2^ *(n = 532)*	1.00	0.62 (0.36–1.07)	0.71 (0.42–1.19)	0.56 (0.29–1.05)	0.2107	0.96 (0.85–1.09)	
Average egg consumption RR^2^ *(n = 532)*	1.00	0.51 (0.29–0.88)	0.53 (0.31–0.89)	0.42 (0.22–0.80)	0.1080	0.96 (0.83–1.11)	
**Low HDL cholesterol** (< 40 mg/dl)							
Multivariable-adjusted OR^1^ *(n = 1115)*	1.00	0.93 (0.66–1.33)	0.82 (0.57–1.16)	0.89 (0.61–1.29)	0.7172		0.98 (0.93–1.05)
Egg consumption at baseline RR^2^ *(n = 498)*	1.00	1.14 (0.69–1.89)	0.65 (0.38–1.16)	0.63 (0.33–1.19)	0.0830		0.92 (0.79–1.07)
Average egg consumption RR^2^ *(n = 498)*	1.00	0.84 (0.50–1.41)	0.52 (0.29–0.92)	0.59 (0.31–1.13)	0.1196		0.93 (0.80–1.09)
***Women***							
**Abdominal obesity** (≥ 85cm)							
Multivariable-adjusted OR^1^ *(n = 1772)*	1.00	1.02 (0.74–1.42)	0.83 (0.57–1.19)	0.68 (0.44–1.05)	0.0458		0.90 (0.81–1.01)
Egg consumption at baseline RR^2^*(n = 725)*	1.00	1.29 (0.85–1.96)	1.28 (0.81–2.02)	1.14 (0.67–1.95)	0.8033		0.97 (0.87–1.09)
Average egg consumption RR^2^ *(n = 725)*	1.00	1.01 (0.64–1.59)	1.24 (0.79–1.97)	0.91 (0.50–1.67)	0.7994		0.94 (0.82–1.07)
**High blood pressure** (≥ 130/85 mmHg)							
Multivariable-adjusted OR^1^ *(n = 1772)*	1.00	1.22 (0.94–1.58)	1.21 (0.91–1.61)	0.97 (0.69–1.35)	0.7589		0.95 (0.87–1.04)
Egg consumption at baseline RR^2^ *(n = 728)*	1.00	1.27 (0.80–2.02)	1.05 (0.60–1.83)	1.23 (0.70–2.17)	0.6206		1.04 (0.94–1.16)
Average egg consumption RR^2^ *(n = 728)*	1.00	0.77 (0.48–1.24)	0.75 (0.45–1.26)	0.90 (0.47–1.70)	0.9386		1.02 (0.88–1.18)
**High fasting blood glucose** (≥ 100 mg/dL)							
Multivariable-adjusted OR^1^ *(n = 1772)*	1.00	0.98 (0.76–1.26)	0.87 (0.66–1.15)	0.75 (0.54–1.04)	0.0641		0.90 (0.83–0.99)
Egg consumption at baseline RR^2^ *(n = 807)*	1.00	1.4 (0.92–2.12)	1.09 (0.69–1.74)	0.84 (0.47–1.51)	0.3060		0.93 (0.82–1.05)
Average egg consumption RR^2^ *(n = 807)*	1.00	0.96 (0.63–1.47)	0.77 (0.48–1.22)	0.74 (0.41–1.35)	0.2684		0.89 (0.76–1.04)
**High triglyceride** (≥ 150 mg/dl)							
Multivariable-adjusted OR^1^ *(n = 1772)*	1.00	1.02 (0.79–1.31)	0.88 (0.66–1.17)	0.89 (0.63–1.24)	0.3631		0.94 (0.86–1.02)
Egg consumption at baseline RR^2^ *(n = 855)*	1.00	0.92 (0.63–1.34)	0.70 (0.47–1.04)	0.85 (0.53–1.34)	0.4090		0.95 (0.85–1.06)
Average egg consumption RR^2^ *(n = 855)*	1.00	0.70 (0.48–1.02)	0.57 (0.39–0.84)	0.62 (0.38–1.02)	0.1486		0.92 (0.80–1.05)
**Low HDL cholesterol** (< 50 mg/dl)							
Multivariable-adjusted OR^1^ *(n = 1772)*	1.00	1.04 (0.80–1.36)	1.05 (0.79–1.41)	0.92 (0.66–1.29)	0.6211		0.97 (0.90–1.05)
Egg consumption at baseline RR^2^ *(n = 438)*	1.00	0.97 (0.63–1.48)	1.18 (0.79–1.78)	1.06 (0.66–1.72)	0.6518		1.04 (0.97–1.11)
Average egg consumption RR^2^ *(n = 438)*	1.00	0.70 (0.46–1.05)	0.64 (0.43–0.94)	0.87 (0.55–1.37)	0.7239		1.01 (0.92–1.10)

*Values are expressed as odds ratios (OR) or relative risk (RR) and 95% confidence intervals.

^1^Multivariable-adjusted OR: Multivariate logistic regression analysis adjusted for age (years), educational level (≥ 12 years, yes or no) and total energy intake (kcal/d) for men and age (year), educational level (≥ 12 years, yes or no), BMI (kg/m2) and total intake energy (kcal/d) for women.

^2^Multivariable-adjusted RR: Multivariate Poisson regression analysis adjusted for age (years), educational level (≥ 12 years, yes or no), regular exercise (regular exercise at least 30 min on three or more days per week, yes or no) and total energy intake (kcal/d) for men and age (year), educational level (≥ 12 years, yes or no), and total intake energy (kcal/d) for women.

^3^*P* values for linear trends were obtained by treating the median value of egg consumption in each category as a continuous value.

In the present study, BMI was significantly associated with the risk of MetS for both men and women (RR = 2.82, 95% CI, 1.87–4.26 for BMI ≥ 23 kg/m^2^ compared to normal weight in men and RR = 2.12, 95% CI, 1.53–2.94 for women, data not shown). In the stratification analysis according to BMI group (< 23 kg/m^2^ or ≥ 23 kg/m^2^), the inverse associations between egg consumption and MetS and/or its components seemed to be stronger in men and women of normal weight (BMI < 23 kg/m^2^). There were significant linear trends for high fasting blood glucose among normal weight men (*P* for trend = 0.0248) and for high TG and MetS among normal weight women (*P* for trend = 0.0198 and *P* for trend = 0.0105, respectively), although egg consumption did not interact significantly with the BMI groups except in the case of high TG in women (*P* for interaction = 0.0288) (Table 5).

**Table 5:** Multivariable-adjusted RRs and 95% CIs of the MetS and its component according to average egg consumption group by BMI category.*

	Weekly egg consumption (No/week)					
	0	0–1	1–3	> 3	*P* trend^1^	*P* interacion
*Men*						
**Metabolic syndrome**						
BMI < 23 kg/m^2^ (n = 333)	1.00	0.44 (0.16–1.16)	0.45 (0.17–1.16)	0.32 (0.09–1.15)	0.2335	0.8701
BMI ≥ 23 kg/m^2^ (n = 342)	1.00	0.60 (0.32–1.10)	0.54 (0.29–0.98)	0.54 (0.28–1.03)	0.2536	
**Abdominal obesity** (≥ 90cm)						
BMI < 23 kg/m^2^ (n = 328)	1.00	0.34 (0.56–1.96)	0.03 (0.05–1.80)	1.25 (0.32–4.83)	0.2535	0.4764
BMI ≥ 23 kg/m^2^ (n = 236)	1.00	0.50 (0.15–1.68)	0.93 (0.31–2.73)	1.03 (0.34–3.12)	0.2593	
**High blood pressure** (≥ 130/85 mmHg)						
BMI < 23 kg/m^2^ (n = 202)	1.00	0.71 (0.29–1.93)	0.85 (0.33–2.20)	0.72 (0.24–2.13)	0.7544	0.1054
BMI ≥ 23 kg/m^2^ (n = 226)	1.00	0.33 (0.16–0.68)	0.25 (0.11–0.58)	0.55 (0.28–1.06)	0.9400	
**High fasting blood glucose** (≥ 100 mg/dL)						
BMI < 23 kg/m^2^ (n = 228)	1.00	0.62 (0.33–1.16)	0.58 (0.32–1.05)	0.32 (0.13–0.78)	0.0248	0.9039
BMI ≥ 23 kg/m^2^ (n = 237)	1.00	0.55 (0.28–1.11)	0.66 (0.36–1.20)	0.43 (0.21–0.87)	0.0770	
**High triglyceride** (≥ 150 mg/dl)						
BMI < 23 kg/m^2^ (n = 265)	1.00	0.45 (0.19–1.08)	0.50 (0.22–1.18)	0.24 (0.07–0.83)	0.0889	0.6012
BMI ≥ 23 kg/m^2^ (n = 267)	1.00	0.62 (0.31–1.23)	0.60 (0.31–1.17)	0.60 (0.28–1.29)	0.5220	
**Low HDL cholesterol** (< 40 mg/dl)	1.00					
BMI < 23 kg/m^2^ (n = 267)	1.00	0.77 (0.42–1.40)	0.37 (0.18–0.78)	0.50 (0.23–1.13)	0.1093	0.4310
BMI ≥ 23 kg/m^2^ (n = 231)	1.00	0.97 (0.35–2.71)	0.77 (0.27–2.20)	0.73 (0.23–2.37)	0.5093	
*Women*						
**Metabolic syndrome**						
BMI < 23 kg/m^2^ (n = 405)	1.00	0.85 (0.43–1.68)	0.66 (0.31–1.43)	0.17 (0.03–0.82)	0.0105	0.1950
BMI ≥ 23 kg/m^2^ (n = 553)	1.00	0.85 (0.56–1.31)	0.78 (0.51–1.21)	0.70 (0.39–1.25)	0.2369	
**Abdominal obesity** (≥ 85cm)						
BMI < 23 kg/m^2^ (n = 386)	1.00	1.00 (0.40–2.44)	1.54 (0.63–3.77)	0.83 (0.24–2.87)	0.8241	0.7924
BMI ≥ 23 kg/m^2^ (n = 339)	1.00	1.07 (0.65–1.75)	1.12 (0.67–1.85)	0.87 (0.45–1.69)	0.5749	
**High blood pressure** (≥ 130/85 mmHg)						
BMI < 23 kg/m^2^ (n = 318)	1.00	0.79 (0.40–1.58)	1.04 (0.45–2.44)	0.34 (0.05–2.42)	0.3624	0.0610
BMI ≥ 23 kg/m^2^ (n = 410)	1.00	0.84 (0.44–1.60)	0.68 (0.34–1.30)	1.03 (0.50–2.14)	0.7837	
**High fasting blood glucose** (≥ 100 mg/dL)						
BMI < 23 kg/m^2^ (n = 333)	1.00	1.07 (0.45–2.55)	1.09 (0.43–2.79	0.55 (0.13–2.34)	0.3874	0.5585
BMI ≥ 23 kg/m^2^ (n = 474)	1.00	1.00 (0.61–1.63)	0.69 0.40–1.17	0.81 (0.42–1.56)	0.4107	
**High triglyceride** (≥ 150 mg/dl)						
BMI < 23 kg/m^2^ (n = 357)	1.00	0.68 (0.36–1.29)	0.60 (0.31–1.18)	0.25 (0.08–0.77)	0.0198	0.0288
BMI ≥ 23 kg/m^2^ (n = 498)	1.00	0.73 (0.46–1.16)	0.56 (0.35–0.90)	0.88 (0.51–1.53)	0.9392	
**Low HDL cholesterol** (< 50 mg/dl)						
BMI < 23 kg/m^2^ (n = 181)	1.00	0.70 (0.35–1.39)	0.62 (0.32–1.21)	0.76 (0.36–1.60)	0.6880	0.9852
BMI ≥ 23 kg/m^2^ (n = 257)	1.00	0.67 (0.40–1.12)	0.62 (0.38–1.01)	0.90 (0.50–1.64)	0.7939	

*Values are expressed as relative risk (RR) and 95% confidence intervals and multivariate Poisson regression analysis adjusted for age (years), educational level (≥ 12 years, yes or no), regular exercise (regular exercise at least 30 min on three or more days per week, yes or no) and total energy intake (kcal/d) for men and age (year), educational level (≥ 12 years, yes or no), and total intake energy (kcal/d) for women.

^1^*P* values for linear trends were obtained by treating the median value of egg consumption in each category as a continuous value.

## Discussion

The present study showed that increased average egg consumption reduced MetS risk in both men and women. Although there was a cross-sectional inverse relationship of egg consumption with abdominal obesity and MetS in men, increased egg consumption was prospectively associated with decreased risk of other components of MetS such as high blood glucose and hypertriglyceridemia, rather than abdominal obesity.

The third Korean National Health and Nutrition Examination Survey reported that individuals aged 30–49 years consumed an average of 3.09 eggs per week, while those aged 50–64 years consumed an average of 1.67 eggs per week, and those ≥ 65 years consumed an average of 0.98 eggs per week [19], which was similar to our data (1.52 eggs/wk for subjects ≥ 40 years old; 2.0 eggs/wk in men; 1.20 eggs/wk in women) collected in a rural community. The Korean annual egg consumption per capita in 2005 was 9.9 kg/person/year and it was relatively low compared with the U.S. (14.6 kg/person/year) and Japan (19 kg/person/year) [20]. It has been recommended that egg consumption be restricted since the early 1970s due to the high cholesterol content of eggs (470 mg/100g) [21]. However, over the last 50 years, a number of cholesterol feeding studies found little impact of dietary cholesterol on blood cholesterol. Adding dietary cholesterol (100mg/day) did not substantially increase lipid profiles: a 2.2 mg/dl increase of total blood cholesterol, a 1.9 mg/dl increase of LDL cholesterol, and a 0.01 increase of the LDL:HDL ratio (from 2.60 to 2.61) [22]. Thus, it seems unlikely that restricting egg consumption would have a beneficial effect on blood lipid profiles. Furthermore, the World Health Organization (WHO, 2003) has also stated that if dairy fat and meat intake are controlled there is no need to severely restrict egg yolk intake [23] and the British Heart Foundation has removed its advice to limit egg consumption to three per week and now recommends eating eggs regularly as part of a balanced and varied diet [24]. In the present study, egg cholesterol contributed 41.3% of total daily dietary cholesterol intake and explained 70.6% of the variation in daily dietary cholesterol. Egg consumption had a non-significant positive relationship with the prevalence of high blood total cholesterol (> 200 mg/dl) (in multivariable-adjusted model for men: RR = 0.99, 95% CI, 0.44–2.24, *P* for trend = 0.9913; for women: RR = 1.16, 95% CI, 0.70–1.93, *P* for trend = 0.5168) (S2 Table).

We found that egg consumption had a beneficial effect on MetS in both prospective and cross-sectional analyses. In the prospective analyses, there were stronger associations with average egg consumption than with baseline egg consumption. This was likely due to reduced intra-individual variation over time [18] and to average consumption reflecting long-term intake which may be etiologically more relevant than baseline value. This beneficial effect of egg consumption on MetS contradicts the standard recommendation of reducing weekly egg consumption as a means of limiting dietary cholesterol and preventing coronary heart disease [22]. To the best of our best knowledge, there have been no epidemiological studies in free-living populations that have explored the relationship between egg consumption and MetS incidence, but several clinical intervention studies have reported an inverse association in individuals with Mets [10, 25].

Among the components of MetS, abdominal obesity, which was inversely correlated with MetS in the cross-sectional analysis, appeared not to be prospectively associated with egg consumption. Although the association was statistically significant for fasting blood glucose and TG only in men, components other than abdominal obesity tended to be inversely associated with average egg consumption. As for TG, an experimental study feeding 3 eggs per day for 12 weeks to overweight white male subjects also showed a significant decrease in plasma TG [26]. In addition, a previous intervention study found that when compared to egg substitutes, whole eggs were associated with improvements in lipoprotein metabolism and inflammation, greater increases in HDL-C, HDL_2_, larger LDL particle diameter, and lecithin cholesterol acyltransferase (LCAT) activity, and greater reductions of VLDL particle diameter [10], plasma tumor necrosis factor α (TNF- α) and serum amyloid A (SAA) [25]. Small LDL particles were highly associated with low HDL-C and high plasma TG [27], and VLDL was associated with risk of MetS [28]. LCAT activity is critically important for HDL particle stability and HDL maturation and thus the increase of LCAT activity with egg consumption may be associated with enhanced HDL-mediated reverse cholesterol transport [29].

Observations of the associations of egg consumption with diabetes [30–32] and of CVD among diabetics [3, 4] have been inconsistent. The observed association with egg consumption differs between the combined data of the Physician’s Health Study and Women’s Health Study [30] and the Kuoppio Ischemic Heart Disease Risk Factor Study [31]. The positive association between egg consumption and the risk of incident diabetes in the case of the combined data of the Physicians’ Health Study and the Women’s Health Study [30] may be due to differences in the dietary sources of cholesterol, such as more red meat, which may increase the risk of diabetes and CVD [33], rather than to differences in egg cholesterol intake, as suggested by a Japanese study [34]. Chronic low-grade inflammation is often seen in patients with MetS and is thought to be a major contributor to insulin resistance [35]. The inverse association between egg consumption and high fasting blood glucose in the present study may be explained by the reduction in inflammation resulting from whole eggs consumption [25] as mentioned above. It was also observed in healthy French subjects [36]. It may be due to flattening of the glycemic and insulin response as a result of improved insulin sensitivity brought about by monounsaturated fatty acids, polyunsaturated fatty acids, and antioxidants including lutein and zeaxanthin and folate in eggs [37–39]. The ability of eggs to lower blood pressure may be explained by the effect of omega-3 fatty acids including eicosa-pentaenoic acid (EPA), which are high in eggs and are known to reduce blood pressure by competing with arachidonic acid in the cyclooxygenase pathway [40]. Although the follow-up time was not long enough and hard outcomes like CVD events were based on self-report in the present study, an non-significant but inverse association between CVD risk using self-reported 97 myocardial infarction or stroke events and egg consumption was found (in the multivariable-adjusted model including confounders selected by the same procedure as in the present study for men: RR = 0.56, 95% CI, 0.25–1.32, P for trend = 0.7035; for women: RR = 0.53, 95% CI, 0.15–1.82, P for trend = 0.1706) (S3 Table).

Several possible mechanisms have been suggested to explain the inverse relationship between egg consumption and components of MetS. However, we cannot rule out the possible effect on MetS of healthy eating patterns associated with egg consumption. Among our subjects, egg consumption tended to be related to diet quality scores such as Mean Adequacy Ratio [41] (*P* for trend 0.001) and Recommended Food Score [42] (*P* for trend 0.001). Therefore, we also adjusted for these dietary quality factors, but this had little impact on the results.

Our study had several limitations. First, the low follow-up rate may have led to biased results, although we found very similar results in a re-analysis among participants who completed all five examinations. Second, our findings may not be generalizable to individuals already suffering from metabolic diseases such as diabetes and hypertension, because the biological effects of egg or dietary cholesterol consumption may differ in our healthy groups (from which existing cases of metabolic disease were excluded). Third, in prospective analyses, metabolic components may fluctuate over time [43], and we did not account for multiple relapses of MetS. However a similar inverse trend was found when we consider hard outcomes like myocardial infarction and stroke (S3 Table). Fourth, this study did not include subjects who consumed extremely high numbers of eggs, and thus we could not draw conclusions about how extremely high egg intake might impact MetS and its components. Finally, although the dietary assessment tool used in this study (FFQ) has been validated, it was not validated for egg consumption. Regardless of these limitations, this study provides insight into the prospective effects of egg consumption on MetS incidence. In particular, the data on average egg consumption should be robust because egg consumption was measured repeatedly over time.

In conclusion, our findings suggest that higher egg consumption may reduce the risk of MetS both in men and women, and the risk of high fasting blood glucose and high triglycerides in men. Current guidelines regarding egg consumption may need to be re-visited and revised for healthy middle-aged and elderly people.

## Supporting Information

S1 Table Age-adjusted characteristics of the study population, according to egg consumption among subjects without MetS. (DOCX)

S2 Table Cross-sectional and prospective association between high total cholesterol (≥ 200 mg/dL) and egg consumption. (DOCX)

S3 Table Multivariable-adjusted RRs and 95% CIs of the cardiovascular disease (CVD) according to average egg consumption group. (DOCX)
