# Supplementary material for: Cross-Sectional and Longitudinal Associations between Egg Consumption and Metabolic Syndrome in Adults ≥ 40 Years Old: The Yangpyeong Cohort of the Korean Genome and Epidemiology Study (KoGES_Yangpyeong)
Source: PLoS One. 2016 Jan 25;11(1):e0147729. doi: 10.1371/journal.pone.0147729 (PMC4726710; doi:10.1371/journal.pone.0147729)
Supplement: S1 Table — (DOCX) [file pone.0147729.s001.docx]

Supporting Information Table 1. Age-adjusted characteristics of the study population, according to egg consumption among subjects without MetS.

|  | Weekly egg consumption (No/week) | | | | |
| --- | --- | --- | --- | --- | --- |
|  | Subjects without MetS *(n=1663)* | | | | |
| Variables | 0 | 0-1 | 1-3 | ˃3 | *P* trend^3^ |
| ***Men (n)*** | 132 | 180 | 196 | 167 |  |
| Age (years) | 64.6±0.88 | 62.2±0.76 | 60.5±0.72 | 60.7±0.78 | 0.0112 |
| Married (%) | 95.8 | 96.7 | 95.7 | 92.7 | 0.0729 |
| Education (≥ High-school, %)^1^ | 21.9 | 27.3 | 37.2 | 45.5 | <.0001 |
| Regular exercise (%)^2^ | 20.9 | 15.1 | 21.2 | 26.2 | 0.0409 |
| Current smoker (%) | 36.7 | 36.4 | 31.3 | 38.2 | 0.5913 |
| Current drinker (%) | 67.2 | 70.7 | 69.8 | 68.3 | 0.8679 |
| BMI (kg/m^2^) | 23.3±0.23 | 23.0±0.2 | 23.2±0.19 | 23.0±0.20 | 0.3761 |
| ***Daily dietary intake*** |  |  |  |  |  |
| Total energy (kcal/d) | 1569.6±40.10 | 1626.3±34.09 | 1787.8±32.74 | 1918.2±35.44 | <.0001 |
| Fiber (g/d) | 16.6±0.45 | 16.5±0.38 | 16.4±0.37 | 17.0±0.40 | 0.3400 |
| Retinol (μg/d) | 31.5±4.52 | 43.2±3.84 | 60±3.69 | 105.7±3.99 | <.0001 |
| β-Carotene (μg/d) | 2375.7±130.13 | 2431.9±110.62 | 2389.2±106.24 | 2657.9±115.0 | 0.0621 |
| Vitamin C (mg/d) | 75.4±3.49 | 76.7±2.97 | 81.3±2.85 | 82.4±3.09 | 0.1267 |
| Cholesterol (mg/d) | 64.6±8.74 | 96.5±7.43 | 145.8±7.13 | 275±7.72 | <.0001 |
| ***Metabolic syndrome components*** |  |  |  |  |  |
| Waist circumference (cm) | 84.6±0.65 | 83.6±0.55 | 83.0±0.53 | 83.5±0.58 | 0.5687 |
| Fasting glucose (mg/dL) | 99.1±1.75 | 100±1.49 | 98.7±1.43 | 99.8±1.54 | 0.8312 |
| Triglycerides (mg/dL) | 125.7±5.93 | 128.1±5.04 | 124.2±4.84 | 125.9±5.24 | 0.9286 |
| HDL cholesterol (mg/dL) | 46.0±0.98 | 46.0±0.83 | 46.3±0.80 | 47.1±0.87 | 0.3084 |
| Systolic blood pressure (mm Hg) | 122.3±1.29 | 120.7±1.09 | 121.6±1.05 | 121.1±1.14 | 0.7430 |
| Diastolic blood pressure (mm Hg) | 78.5±0.85 | 77.7±0.72 | 78.0±0.69 | 78.0±0.75 | 0.9034 |
|  |  |  |  |  |  |
| ***Women (n)*** | 282 | 244 | 263 | 169 |  |
| Age (years) | 61.1±0.60 | 56.8±0.64 | 53.8±0.62 | 52.6±0.77 | <.0001 |
| Married (%) | 80.3 | 84.0 | 85.6 | 77.3 | 0.3122 |
| Education (≥ High-school, %)^1^ | 25.2 | 26.1 | 38.3 | 43 | <.0001 |
| Regular exercise (%)^2^ | 25.3 | 23.1 | 26.4 | 29.0 | 0.2661 |
| Current smoker (%) | 3.9 | 3.3 | 1.5 | 2.4 | 0.2850 |
| Current drinker (%) | 34.8 | 33.4 | 38.4 | 35.7 | 0.6528 |
| BMI (kg/m^2^) | 23.7±0.18 | 23.9±0.18 | 23.4±0.18 | 23.6±0.22 | 0.3224 |
| ***Daily dietary intake*** |  |  |  |  |  |
| Total energy (kcal/d) | 1405.9±26.25 | 1443.4±27.38 | 1549.2±26.63 | 1653.6±33.34 | <.0001 |
| Fiber (g/d) | 15.4±0.28 | 15.1±0.29 | 14.7±0.28 | 14.6±0.36 | 0.0849 |
| Retinol (μg/d) | 32.9±3.28 | 43.5±3.42 | 59.1±3.33 | 88.2±4.17 | <.0001 |
| β-Carotene (μg/d) | 2464.9±86.69 | 2408.8±90.43 | 2247.5±87.96 | 2336.6±110.12 | 0.2977 |
| Vitamin C (mg/d) | 83.0±2.54 | 85.4±2.65 | 81.4±2.58 | 82.6±3.22 | 0.6867 |
| Cholesterol (mg/d) | 60.1±5.57 | 82.5±5.81 | 127.6±5.65 | 223.8±7.07 | <.0001 |
| ***Metabolic syndrome components*** |  |  |  |  |  |
| Waist circumference (cm) | 80.8±0.49 | 81.2±0.51 | 79.6±0.50 | 79.6±0.62 | 0.0376 |
| Fasting glucose (mg/dL) | 94.6±1.03 | 95.0±1.07 | 93.9±1.04 | 92.3±1.30 | 0.1144 |
| Triglycerides (mg/dL) | 107.4±2.75 | 109.6±2.86 | 106.0±2.79 | 104.8±3.49 | 0.3939 |
| HDL cholesterol (mg/dL) | 49.0±0.67 | 48.3±0.70 | 49.0±0.68 | 49.4±0.85 | 0.5721 |
| Systolic blood pressure (mm Hg) | 114.8±0.89 | 116.9±0.93 | 117±0.90 | 116.8±1.13 | 0.2817 |
| Diastolic blood pressure (mm Hg) | 75.2±0.56 | 75.6±0.59 | 75.4±0.57 | 74.6±0.71 | 0.4062 |

All values were adjusted for age and expressed as Mean±SE or percent.

All nutrients were energy-adjusted except for total energy intake.

^1^≥ High School graduates (12 years of education).

^2^≥ 3 times/week and 30 min/session.

^3^*P*-values for linear trends were obtained using the general linear model (GLM).
