# Supplementary material for: Cross-Sectional and Longitudinal Associations between Egg Consumption and Metabolic Syndrome in Adults ≥ 40 Years Old: The Yangpyeong Cohort of the Korean Genome and Epidemiology Study (KoGES_Yangpyeong)
Source: PLoS One. 2016 Jan 25;11(1):e0147729. doi: 10.1371/journal.pone.0147729 (PMC4726710; doi:10.1371/journal.pone.0147729)
Supplement: S2 Table — (DOCX) [file pone.0147729.s002.docx]

Supporting Information Table 2. Cross-sectional and longitudinal association between high total cholesterol and egg consumption.*

|  | Weekly egg consumption (No/week) | | | | |
| --- | --- | --- | --- | --- | --- |
|  | 0 | 0-1 | 1-3 | > 3 | *P* trend^3^ |
| ***Men*** |  |  |  |  |  |
| Cross-sectional analysis (n=1115) |  |  |  |  |  |
| Prevalent cases at baseline / No. of subjects | 83 / 229 | 103 / 296 | 120 / 320 | 109 / 270 |  |
| Median intake (min-max) | 0 | 0.58 (0.16-0.86) | 1.50 (1.50-2.25) | 5.25 (3.50-31.5) |  |
| Age-adjusted OR | 1.00 | 0.93 (0.65-1.33) | 1.03 (0.72-1.47) | 1.16 (0.81-1.68) | 0.2293 |
| Multivariable-adjusted OR^1^ | 1.00 | 0.93 (0.65-1.34) | 1.03 (0.72-1.48) | 1.13 (0.78-1.65) | 0.3376 |
| Longitudinal analysis (n=450) |  |  |  |  |  |
| Egg consumption at baseline |  |  |  |  |  |
| No. of cases / person years | *10 / 272* | *21 / 373* | *28 / 454* | *16 / 313* |  |
| Median intake (min-max) | 0 | 0.58 (0.12-0.86) | 1.50 (1.50-2.25) | 3.50 (3.50-21.0) |  |
| Age-adjusted RR | 1.00 | 1.19 (0.72-3.07) | 1.61 (0.80-3.22) | 1.31 (0.61-2.79) | 0.7977 |
| Multivariable-adjusted RR^2^ | 1.00 | 1.50 (0.72-3.11) | 1.64 (0.81-3.34) | 1.35 (0.60-3.02) | 0.7567 |
| Average egg consumption |  |  |  |  |  |
| No. of cases / person years | *9 / 188* | *22 / 429* | *28 / 502* | *16 / 293* |  |
| Median intake (min-max) | 0 | 0.58 (0.12-0.87) | 1.50 (1.04-2.88) | 3.70 (3.04-21.0) |  |
| Age-adjusted RR | 1.00 | 0.98 (0.47-2.07) | 1.07 (0.52-2.20) | 1.01 (0.46-2.20) | 0.9526 |
| Multivariable-adjusted RR^2^ | 1.00 | 0.97 (0.46-2.06) | 1.06 (0.51-2.20) | 0.99 (0.44-2.24) | 0.9913 |
| ***Women*** |  |  |  |  |  |
| Cross-sectional analysis (n=1772) |  |  |  |  |  |
| Prevalent cases at baseline / No. of subjects | 332 / 595 | 267 / 495 | 184 / 418 | 132 / 264 |  |
| Median intake (min-max) | 0 | 0.58 (0.12-0.86) | 1.50 (1.50-2.25) | 3.50 (3.50-31.5) |  |
| Age-adjusted OR | 1.00 | 1.03 (0.81-1.32) | 0.75 (0.58-0.98) | 1.00 (0.74-1.35) | 0.5617 |
| Multivariable-adjusted OR^1^ | 1.00 | 1.02 (0.80-1.31) | 0.78 (0.60-1.02) | 1.01 (0.74-1.38) | 0.7282 |
| Longitudinal analysis (n=515) |  |  |  |  |  |
| Egg consumption at baseline |  |  |  |  |  |
| No. of cases / person years | *34 / 440* | *36 / 367* | *43 / 480* | *27 / 284* |  |
| Median intake (min-max) | 0 | 0.58 (0.12-0.86) | 1.50 (1.50-2.25) | 3.50 (3.50-14.0) |  |
| Age-adjusted RR | 1.00 | 1.31 (0.85-2.01) | 1.22 (0.80-1.87) | 1.31 (0.81-2.11) | 0.3904 |
| Multivariable-adjusted RR^2^ | 1.00 | 1.29 (0.84-1.99) | 1.17 (0.76-1.80) | 1.24 (0.76-2.01) | 0.5710 |
| Average egg consumption |  |  |  |  |  |
| No. of cases / person years | *29 / 342* | *39 / 454* | *45 / 506* | *27 / 269* |  |
| Median intake (min-max) | 0 | 0.40 (0.12-0.87) | 1.50 (1.04-2.75) | 3.62 (3.04-14.0) |  |
| Age-adjusted RR | 1.00 | 1.04 (0.66-1.62) | 1.10 (0.71-1.72) | 1.27 (0.77-2.09) | 0.3010 |
| Multivariable-adjusted RR^2^ | 1.00 | 1.02 (0.65-1.60) | 1.07 (0.69-1.66) | 1.16 (0.70-1.93) | 0.5168 |

*Values are expressed as odds ratios (OR) or relative risk (RR) and 95% confidence intervals.

^1^Multivariable-adjusted OR: Multivariate logistic regression analysis adjusted for age (years), educational level (≥ 12 years, yes or no) and total energy intake (kcal/d) for men and age (year), educational level (≥ 12 years, yes or no), BMI (kg/m2) and total intake energy (kcal/d) for women.

^2^Multivariable-adjusted RR: Multivariate Poisson regression analysis adjusted for age (years), educational level (≥ 12 years, yes or no), regular exercise (regular exercise at least 30 min on three or more days per week, yes or no) and total energy intake (kcal/d) for men and age (year), educational level (≥ 12 years, yes or no), and total intake energy (kcal/d) for women.

^3^*P* values for linear trends were obtained by treating the median value of egg consumption in each category as a continuous value.
