# Supplementary material for: Cross-Sectional and Longitudinal Associations between Egg Consumption and Metabolic Syndrome in Adults ≥ 40 Years Old: The Yangpyeong Cohort of the Korean Genome and Epidemiology Study (KoGES_Yangpyeong)
Source: PLoS One. 2016 Jan 25;11(1):e0147729. doi: 10.1371/journal.pone.0147729 (PMC4726710; doi:10.1371/journal.pone.0147729)
Supplement: S3 Table — (DOCX) [file pone.0147729.s003.docx]

Supporting Information Table 3. Multivariable-adjusted RRs and 95% CIs of the cardiovascular disease (CVD) according to average egg consumption group.*

|  | Weekly egg consumption (No/week) | | | | |
| --- | --- | --- | --- | --- | --- |
|  | 0 | 0-1 | 1-3 | > 3 | *P* trend^1^ |
| ***Men*** *(n=1123)* |  |  |  |  |  |
| No. of cases / person years | 12/713 | 11/1476 | 8/1756 | 9/1200 |  |
| Egg consumption at baseline |  |  |  |  |  |
| Multivariable-adjusted RR | 1.00 | 0.62 (0.27-1.38) | 0.43 (0.19-0.99) | 0.42 (0.16-1.09) | 0.1846 |
| Average egg consumption |  |  |  |  |  |
| Multivariable-adjusted RR | 1.00 | 0.44 (0.18-1.07) | 0.34 (1.14-0.81) | 0.56 (0.24-1.32) | 0.7035 |
| ***Women*** *(n=1781)* |  |  |  |  |  |
| No. of cases / person years | 18/2033 | 22/2708 | 14/2293 | 3/1131 |  |
| Egg consumption at baseline |  |  |  |  |  |
| Multivariable-adjusted RR | 1.00 | 1.13 (0.61-2.10) | 1.18 (0.57-2.42) | 0.89 (0.34-2.33) | 0.8834 |
| Average egg consumption |  |  |  |  |  |
| Multivariable-adjusted RR | 1.00 | 1.13 (0.60-2.13) | 1.07 (0.53-2.17) | 0.53 (0.15-1.82) | 0.1706 |

*Values are expressed as relative risk (RR) and 95% confidence intervals and multivariable-adjusted RR was adjusted for age (years), educational level (≥ 12 years, yes or no), regular exercise (regular exercise at least 30 min on three or more days per week, yes or no) and total energy intake (kcal/d) for men and age (year), educational level (≥ 12 years, yes or no), and total intake energy (kcal/d) for women.

^1^*P* values for linear trends were obtained by treating the median value of egg consumption in each category as a continuous value.
